# Optimizing Tissue Preparation for Molecular Testing in Thyroid Tumors: A Retrospective Analysis of 177 Cases from a High-Volume Japanese Center

**DOI:** 10.3390/cancers18182975

**Published:** 2026-09-15

**Authors:** Mitsuyoshi Hirokawa, Miyoko Higuchi, Ayana Suzuki, Minoru Kihara, Takashi Akamizu

**Affiliations:** 1 Department of Diagnostic Pathology and Cytology, Kuma Hospital, 8-2-35 Shimoyamate-dori, Chuo-ku, Kobe 650-0011, Hyogo, Japan; higuchi01@kuma-h.or.jp (M.H.); suzuki01@kuma-h.or.jp (A.S.); 2 Department of Surgery, Kuma Hospital, 8-2-35 Shimoyamate-dori, Chuo-ku, Kobe 650-0011, Hyogo, Japan; kihara@kuma-h.or.jp; 3 Department of Internal Medicine, Kuma Hospital, 8-2-35 Shimoyamate-dori, Chuo-ku, Kobe 650-0011, Hyogo, Japan; akamizu@kuma-h.or.jp

**Keywords:** thyroid neoplasms, companion diagnostics, genetic testing, NGS assay performance, PCR-based diagnostics, specimen handling, fixation, formalin-fixed paraffin-embedded tissue, high-throughput nucleotide sequencing

## Abstract

**Background/Objectives**: Reliable molecular testing is essential for selecting targeted therapies for thyroid tumors; however, assay performance can be affected by pre-analytical tissue conditions. This retrospective, single-center study evaluated real-world formalin-fixed, paraffin-embedded (FFPE) specimens to identify pathology practices associated with successful companion diagnostic testing using the next-generation sequencing (NGS)-based Oncomine Dx assay and the polymerase chain reaction (PCR)-based BRAF3 assay widely used in routine clinical care in Japan. **Methods**: We retrospectively analyzed 177 cases with thyroid tumors tested at a high-volume thyroid center in Japan between 2022 and 2026. **Results**: All 79 specimens analyzed using the BRAF3 assay yielded successful test results, including specimens fixed in unbuffered formalin and archival blocks stored for up to 27 years. In contrast, the Oncomine Dx assay was successful in 93 of 98 specimens (94.9%), with all five failures occurring in specimens fixed in unbuffered formalin. DNA failure was more common than RNA failure. All five Oncomine Dx failures occurred in archived tissue blocks stored for 3–9 years, all of which had been fixed in unbuffered formalin. The BRAF3 test had a shorter turnaround time and was more frequently used in rapidly progressive tumors, such as anaplastic thyroid carcinoma. **Conclusions**: The NGS-based Oncomine Dx assay showed reduced success in samples stored long-term or in unbuffered specimens, whereas RNA preservation was high. In contrast, the PCR-based BRAF3 assay remained highly reliable even in long-stored archival blocks. Because fixative type and storage duration were confounded in this cohort, the independent effects of these variables could not be determined. The study’s findings provide practical real-world insights into how specimen handling, fixation, and storage conditions may influence the reliability of genomic diagnostics in routine practice.

## Simple Summary

Reliable genetic testing is increasingly important for selecting appropriate treatment for thyroid tumors. However, the handling of tissue samples before analysis can greatly affect the test results. We examined samples from a high-volume thyroid center in Japan to determine how routine pathology practices affect the quality of two genetic tests used in clinical care. One test remained reliable even with older or less optimally processed samples, whereas the other was more sensitive to tissue fixation and storage methods. Additionally, rapid preservation of tissue after surgery helped maintain the quality of the genetic material needed for testing. These results provide practical real-world insights that can help pathology laboratories preserve sample quality, improve the reliability of genetic testing, and support treatment decisions for patients with thyroid tumors.

## 1. Introduction

Recent advances in molecular-targeted therapies have transformed the clinical management of thyroid tumors, as actionable genomic alterations, including *BRAF* variants, *RET* pathogenic variants or *RET* fusions, and *NTRK* fusions, increasingly guide treatment selection [1,2,3,4,5]. Comprehensive genomic profiling in Japan has confirmed these alterations as important oncologic drivers and therapeutic targets, supporting the use of BRAF/MEK inhibitors for tumors harboring *BRAF* V600E variants and RET or tropomyosin receptor kinase (TRK) inhibitors for fusion-positive disease [6,7,8,9,10].

Molecular testing has become an essential component of precision oncology for thyroid tumors. In Japan, reimbursed companion diagnostics include two platforms with distinct analytical scopes. Oncomine Dx Target Test Multi-CDx System (Oncomine Dx: Thermo Fisher Scientific, Waltham, MA, USA) is the next-generation sequencing (NGS)-based assay that provides broad molecular profiling, detecting *BRAF* variants as well as *RET* point mutations and fusions [11,12]. In contrast, MEBGEN BRAF3 test (BRAF3: Medical & Biological Laboratories Co., Ltd., Nagoya, Japan) is a targeted polymerase chain reaction (PCR)-based assay designed solely to identify *BRAF* V600E [13,14]. This fundamental difference in analytical breadth underlies their complementary clinical roles and is central to interpreting assay performance in real-world practice.

Pre-analytical conditions of formalin-fixed paraffin-embedded (FFPE) tissue—such as handling, fixation, sampling, and block age—can affect DNA and RNA integrity and influence the success of molecular assays [15,16,17,18,19,20]. However, the impact of routine pathology practices and long-term specimen storage on the performance of companion diagnostics for thyroid tumors remains insufficiently reported in real-world settings. Since companion diagnostic testing became reimbursable in Japan in 2022, our institution has used both Oncomine Dx and BRAF3, allowing evaluation of real-world FFPE specimens and their association with assay success. Therefore, we retrospectively analyzed 177 thyroid tumor cases to assess how pre-analytical tissue conditions relate to the performance of these assays, recognizing the potential to identify associations rather than specific practices that independently improve assay outcomes.

## 2. Materials and Methods

### 2.1. Ethical Considerations

The study protocol was approved by the Institutional Review Board of Kuma Hospital on 12 October 2023 (approval no. 20231012-4), and the procedures were performed in accordance with the tenets of the 1964 Declaration of Helsinki and its later amendments. All participants provided informed consent.

### 2.2. Case Selection

Between August 2022 and April 2026, 192 patients with thyroid tumors underwent companion diagnostic testing at our institution. To minimize variability in pre-analytical tissue handling, 15 patients whose FFPE blocks had been prepared at outside institutions were excluded, leaving 177 patients for analysis. Clinical data were obtained from electronic medical records. Pathological diagnoses were established by consensus between two board-certified pathologists specializing in thyroid pathology. All 192 patients represented consecutive cases for whom companion diagnostic testing was requested during the study period. No additional clinical or technical selection criteria were applied, and the only exclusion criterion was preparation of FFPE blocks at outside institutions.

### 2.3. Fixation and Tissue Processing

Specimens were processed using one of two fixatives according to the institutional practice in place at the time of tissue collection. Until April 2020, tissues were fixed in unbuffered formalin (Ufix^®^, Sakura Finetek Japan Co., Ltd., Tokyo, Japan); thereafter, 10% neutral buffered formalin (NBF) (Muto Pure Chemicals Co. Ltd., Tokyo, Japan) was used. Of the specimens analyzed, 116 were fixed in 10% NBF and 61 in unbuffered formalin. All specimens stored for >6 years had been fixed in unbuffered formalin, reflecting historical practices. Because the fixative type changed over time, fixative type and storage duration were inherently confounded in this cohort. All specimens stored for more than 6 years had been fixed in unbuffered formalin. This methodological limitation directly affects interpretation of assay performance.

Fixation was performed immediately after resection. At our institution, all thyroidectomy specimens are delivered to pathology within 30 min of removal, and formalin fixation is consistently started within 1 h. Throughout the study period, formalin injection into the thyroid parenchyma using a 22-gauge needle was performed for all thyroidectomy specimens prior to standard immersion fixation. Surgical specimens were fixed for 48–72 h and biopsy specimens for 24 h. During gross examination, at least one noncalcified block was prepared for each case; consequently, all specimens included in the analysis were nondecalcified FFPE tissues.

### 2.4. Genetic Testing

Genetic testing was performed at certified commercial laboratories. Oncomine Dx uses both DNA and RNA to detect *BRAF* V600E and *RET* variants, as well as *RET* fusion genes. Testing was conducted by LSI Medience Corporation (Tokyo, Japan). A minimum tumor cell content of 30% was required. To meet this criterion, tumor-rich regions were selected for macrodissection while avoiding necrotic, fibrotic, and dense inflammatory infiltrate-rich regions. BRAF3 detects *BRAF* V600E variants. Testing was performed by SRL, Inc. (Tokyo, Japan). For both assays, success or failure was determined according to the quality control criteria used by the respective certified laboratories. Detailed internal thresholds for Oncomine Dx were not available to our institution because the assay is performed externally. However, laboratory reports indicated that DNA and RNA quality metrics (e.g., DNA-AQ20 and DNA% reads) were used to assess analyzable nucleic acid input. Cases were classified as DNA failure or RNA failure when the corresponding library did not meet the laboratory’s predefined quality requirements for sequencing or fusion analysis.

Among the 177 patients included in the study, 98 underwent Oncomine Dx and 79 underwent BRAF3. Of these, 35 were evaluated using both assays. Because only 35 patients underwent both assays, this study does not represent a paired or head-to-head comparison between Oncomine Dx and BRAF3. In routine clinical practice at our institution, assay selection was determined by clinical context: BRAF3 was often chosen when rapid *BRAF* V600E assessment was needed, whereas Oncomine Dx was selected when broader molecular profiling was clinically indicated.

Because only five Oncomine Dx failures occurred and fixative type and FFPE storage duration were strongly confounded, meaningful inferential statistical analyses (including regression modeling or comparative testing) were not feasible. Therefore, the statistical evaluation was descriptive.

## 3. Results

The histological distribution of the tumors tested on each platform is summarized in Table 1. Papillary thyroid carcinoma was the most common tumor type (138/177, 78.0%) and predominated in both assay groups. Anaplastic thyroid carcinomas were more frequent in the BRAF3 group (15/79, 19.0%) than in the Oncomine Dx group (8/98, 8.2%), suggesting that clinical urgency may have influenced assay selection. Although the testing laboratories listed longer official TATs—20 days for Oncomine Dx and 14 days for BRAF3—, the actual TATs observed in our cohort were shorter. The median TAT for Oncomine Dx was 10 days (range: 7–17 days), whereas that for BRAF3 was 8 days (range: 7–17 days). These observations indicate that BRAF3 was often used in settings requiring rapid results, but the study cannot determine whether turnaround time directly influenced assay choice.

Table 2 summarizes assay success according to FFPE block storage duration and fixative type. Most specimens had been stored for ≤6 years at the time of testing (84.7% for Oncomine Dx cases and 70.9% for BRAF3), although archival blocks stored for up to 18 and 27 years, respectively, were also analyzed. All 79 specimens tested with BRAF3 were successfully analyzed, including long-stored specimens and those fixed in unbuffered formalin. In contrast, Oncomine Dx was successful in 93 of 98 specimens (94.9%). All five failures occurred in specimens fixed in unbuffered formalin, but these specimens were also disproportionately older, making it impossible to distinguish the effects of fixative type from storage duration. This was unexpected because RNA assays are generally more prone to failure in aged FFPE samples. Among the 35 specimens tested with both assays, all yielded successful results for both Oncomine Dx and BRAF3. No discordant findings were observed. Because no failures occurred in this subgroup, further comparative statistical analysis was not informative.

The clinicopathological and preanalytical characteristics of the five cases in which Oncomine Dx testing failed are summarized in Table 3. All five specimens were fixed in unbuffered formalin; four were fixed for 48 h and one for 72 h. Tumor cell content exceeded 30% in all five specimens, with no necrosis or inflammatory cell infiltration, and specimens were derived from nondecalcified blocks. FFPE block storage duration ranged from 3 to 9 years. The RNA-failure case had been stored for 3–4 years, whereas the four DNA-failure cases had been stored for 6–9 years. These observations describe the conditions under which failures occurred but do not determine whether fixative type, storage duration, or their combination was primarily responsible. Among the DNA-failure cases, DNA-AQ20 values ranged from 62 to 96, whereas DNA% read was consistently low (0.51–5.58%). The RNA-failure case showed adequate mappable reads but insufficient RNA quality for successful fusion analysis.

DNA-AQ20: Average length of regions with base quality ≥ 20; higher values indicate less DNA fragmentation (≥90 acceptable).

DNA% Reads: Proportion of reads derived from the DNA library; reflects adequacy of DNA input for analysis (≥0.7 acceptable).

## 4. Discussion

This study evaluated pre-analytical factors influencing the success of companion diagnostic testing in 177 FFPE thyroid tumor specimens processed at a high-volume thyroid center. All specimens tested with the BRAF3 assay were successfully analyzed, whereas the Oncomine Dx assay had a success rate of 94.9%. All five assay failures occurred in the Oncomine Dx group in specimens fixed in unbuffered formalin, and four DNA-failure cases involved blocks stored for 6–9 years. These observations indicate an association between assay performance, fixative type, and storage duration. However, the study cannot determine whether failure was primarily related to unbuffered formalin, storage duration, or their combined effect. The findings highlight the importance of appropriate tissue processing for reliable molecular testing.

Fixative type appeared to be an important factor associated with Oncomine Dx performance. All five unsuccessful specimens were fixed in unbuffered formalin, whereas no failures occurred among specimens fixed in 10% NBF. Unbuffered formalin can promote nucleic acid fragmentation and acid-induced depurination, particularly during prolonged storage [21,22]. Our findings are therefore consistent with previous evidence that buffered formalin better preserves nucleic acid integrity for molecular testing. However, because all specimens stored for >6 years in our cohort had been fixed in unbuffered formalin, the independent effects of fixative type and storage duration could not be determined.

Storage duration may have contributed to Oncomine Dx failure, as all five failures occurred in blocks stored for 3–9 years, including four DNA-failure cases stored for 6–9 years. DNA degradation during prolonged FFPE storage has been reported [17,23,24,25,26], whereas RNA integrity is more sensitive to pre-fixation conditions such as cold ischemia [27,28]. In our institution, fixation is initiated within 1 h of resection using an injection-based method, which may have helped preserve RNA; however, this explanation remains speculative because cold ischemia time and other pre-analytical variables were not systematically assessed. The predominance of DNA failure in older blocks should therefore be interpreted cautiously. Overall, these observations underscore the importance of controlling both pre-fixation handling and long-term storage of tissue intended for NGS-based testing.

This study suggests that differences in assay design—namely a rapid single-target PCR assay versus a broader NGS-based companion diagnostic—contribute to the observed patterns in assay performance and clinical use. BRAF3 successfully analyzed all specimens, including long-stored archival blocks and those fixed in unbuffered formalin, consistent with the tolerance of short-amplicon PCR assays to nucleic acid fragmentation. The shorter turnaround time of BRAF3 may have contributed to its more frequent use in anaplastic thyroid carcinoma; however, the study cannot establish whether turnaround time directly influenced assay selection. These findings should not be interpreted as evidence of overall superiority of BRAF3 over Oncomine Dx. The assays differ fundamentally in target breadth, analytical requirements, and intended clinical roles. BRAF3 may be useful when rapid *BRAF* V600E detection or testing of older or suboptimally fixed specimens is needed, whereas Oncomine Dx remains important when broader molecular characterization is required.

This study has several limitations. First, it was conducted at a single institution, and tissue-processing practices, including the historical use of unbuffered formalin and rapid injection-based fixation, may limit the generalizability of the findings. Second, assay selection was retrospective and clinically driven, resulting in non-equivalent patient populations and only 35 patients tested with both assays. Third, only five Oncomine Dx failures occurred, precluding robust statistical evaluation of individual preanalytical factors. In particular, fixative type and storage duration were partly confounded because all specimens stored for >6 years had been fixed in unbuffered formalin. Fourth, detailed information on ischemic time and tissue thickness was not available for all specimens, preventing direct evaluation of their effects on nucleic acid quality. These limitations should be considered when extrapolating the findings to other oncology centers. Larger multicenter studies with standardized preanalytical data are needed to determine the independent effects of fixation, storage duration, and other tissue-processing variables on assay performance.

## 5. Conclusions

This study provides practical, pathology-centered insights into specimen handling and assay selection for companion diagnostic testing of thyroid tumors. Oncomine Dx failures were observed only in specimens fixed in unbuffered formalin, whereas all BRAF3 assays were completed, including those using long-term archival blocks. These findings suggest that fixation conditions may influence assay performance, although independent effects of fixation method or storage duration cannot be determined from this cohort. Rapid initiation of fixation may contribute to RNA preservation; however, this mechanism was not directly evaluated and should be regarded as hypothesis-generating.

While our observations may help inform practical considerations, they do not establish a general clinical algorithm for assay selection. Buffered formalin and careful preanalytical handling remain advisable for reliable molecular testing. Assay choice should be individualized based on specimen quality and clinical needs, with BRAF3 potentially useful for rapid *BRAF* V600E testing or older or suboptimally fixed specimens, and Oncomine Dx for broader molecular profiling when conditions permit. Overall, integrating appropriate specimen processing with assay selection may improve the reliability and clinical utility of molecular diagnostics for thyroid tumors.

## Figures and Tables

**Table 1:** Histological types of thyroid tumors submitted for companion diagnostic testing.

Histological Type	Oncomine Dx Assay *n* = 98 (%)	BRAF3 Assay *n* = 79 (%)	Total *n* = 177 (%)
PTC	77 (78.6%)	61 (77.2%)	138 (78.0%)
FTC	5 (5.1%)	1 (1.3%)	6 (3.4%)
MTC	3 (3.1%)	0 (0%)	3 (1.7%)
PDTC	5 (5.1%)	2 (2.5%)	7 (4.0%)
ATC	8 (8.2%)	15 (19.0%)	23 (13.0%)

PTC, papillary thyroid carcinoma; FTC, follicular thyroid carcinoma; MTC, medullary thyroid carcinoma; PDTC, poorly differentiated thyroid carcinoma; ATC, anaplastic thyroid carcinoma.

**Table 2:** Genetic testing success rates by fixative type and FFPE block storage duration.

FFPE Storage Duration (Years)	Fixative	Oncomine Dx (*n* = 98)		**BRAF3 (*n* = 79)** Success/Failure/Success Rate
		DNA Success/Failure/Success Rate	RNA Success/Failure/Success Rate	
<1	10% NBF	38/0/100%	38/0/100%	27/0/100%
1–2	10% NBF	14/0/100%	14/0/100%	4/0/100%
2–3	10% NBF	6/0/100%	6/0/100%	6/0/100%
	Unbuffered	1/0/100%	1/0/100%	
3–4	10% NBF	9/0/100%	9/0/100%	4/0/100%
	Unbuffered	2/0/100%	1/1/50.0%	1/0/100%
4–5	10% NBF	3/0/100%	3/0/100%	4/0/100%
	Unbuffered	2/0/100%	2/0/100%	3/0/100%
5–6	10% NBF			1/0/100%
	Unbuffered	8/0/100%	8/0/100%	6/0/100%
6–7	Unbuffered	5/2/28.6%	7/0/100%	2/0/100%
7–8	Unbuffered	1/1/50%	2/0/100%	
8–9	Unbuffered	0/1/0%	1/0/100%	4/0/100%
9–10	Unbuffered	2/0/100%	2/0/100%	4/0/100%
10–20	Unbuffered	3/0/100%	3/0/100%	12/0/100%
21–27	Unbuffered			1/0/100%

FFPE, formalin-fixed paraffin-embedded; NBF, neutral buffered formalin.

**Table 3:** Clinicopathological and pre-analytical characteristics of the five unsuccessful Oncomine Dx specimens.

Case	Age/Sex	Site	Tumor	Failed Nucleic Acid	DNA-AQ20	DNA% Read	RNA Mappable Reads (Count)	FFPE Storage (Years)	Tumor Size (mm)	Fixation Time (h)	Fixative	Tumor Cell Content	Decalcification	Necrosis	Inflammatory Cells
1	23/female	Thyroid	PTC	DNA	73	5.01	120,984	6–7	10	48	Unbuffered formalin	>30%	None	None	None
2	79/female	Thyroid	FTC	DNA	62	0.51	21,466	8–9	66	72	Unbuffered formalin	>30%	None	None	None
3	76/female	Neck	PTC	DNA	90	2.65	85,984	6–7	12	48	Unbuffered formalin	>30%	None	None	None
4	58/male	Thyroid	PTC	DNA	95	5.58	141,423	7–8	46	48	Unbuffered formalin	>30%	None	None	None
5	71/female	Lymph node	ATC	RNA	96	2.22	76,127	3–4	27	48	Unbuffered formalin	>30%	None	None	None

Failed nucleic acid: analyte that did not meet quality criteria. PTC, papillary thyroid carcinoma; FTC, follicular thyroid carcinoma; ATC, anaplastic thyroid carcinoma.

## Data Availability

The data presented in this study are not publicly available owing to patient privacy and ethical restrictions. De-identified data may be made available from the corresponding author upon reasonable request and with approval from the Institutional Review Board of Kuma Hospital.

## Abbreviations

The following abbreviations are used in this manuscript:

BRAF3	MEBGEN BRAF3 test
FFPE	formalin-fixed, paraffin-embedded
NGS	next-generation sequencing
Oncomine Dx	Oncomine Dx Target Test Multi-CDx System
PCR	polymerase chain reaction
TRK	tropomyosin receptor kinase
